# Quality of life, psychological burden, needs, and satisfaction during specialized inpatient palliative care in family caregivers of advanced cancer patients

**DOI:** 10.1186/s12904-017-0206-z

**Published:** 2017-05-10

**Authors:** Anneke Ullrich, Lilian Ascherfeld, Gabriella Marx, Carsten Bokemeyer, Corinna Bergelt, Karin Oechsle

**Affiliations:** 1 Department of Oncology, Haematology and Bone Marrow Transplant, Palliative Care Unit, University Medical Center Hamburg-Eppendorf, Martinistr. 52, 20246 Hamburg, Germany; 2 Department of Medical Psychology, University Medical Center Hamburg-Eppendorf, Martinistr. 52, 20246 Hamburg, Germany; 3 Department of Palliative Medicine, University Medical Center Göttingen, Von-Siebold-Str. 3, 37075 Göttingen, Germany

**Keywords:** Family caregiver, Cancer, Palliative care, Quality of life, Psychological burden, Supportive needs

## Abstract

**Background:**

This pilot study aimed to investigate quality of life, psychological burden, unmet needs, and care satisfaction in family caregivers of advanced cancer patients (FCs) during specialized inpatient palliative care (SIPC) and to test feasibility and acceptance of the questionnaire survey.

**Methods:**

During a period of 12 weeks, FCs were recruited consecutively within 72 h after the patient’s admission. They completed validated scales on several outcomes: quality of life (SF-8), distress (DT), anxiety (GAD-7), depression (PHQ-9), supportive needs (FIN), palliative care outcome (POS), and satisfaction with care (FAMCARE-2). We used non-parametric tests, *t*-tests and correlation analyses to address our research questions.

**Results:**

FCs showed high study commitment: 74 FCs were asked to participate whereof 54 (73%) agreed and 51 (69%) returned the questionnaire. Except for “bodily pain”, FCs’ quality of life (SF-8) was impaired in all subscales. Most FCs (96%) reported clinically significant own distress (DT), with sadness, sorrows and exhaustion being the most distressing problems (80–83%). Moderate to severe anxiety (GAD-7) and depression (PHQ-9) were prevalent in 43% and 41% of FCs, respectively. FCs scored a mean number of 16.3 of 20 needs (FIN) as very or extremely important (SD 3.3), 20% of needs were unmet in >50% of FCs. The mean POS score assessed by FCs was 16.6 (SD 5.0) and satisfaction (FAMCARE-2) was high (73.4; SD 8.3).

**Conclusions:**

This pilot study demonstrated feasibility of the questionnaire survey and showed relevant psychosocial burden and unmet needs in FCs during SIPC. However, FCs’ satisfaction with SIPC seemed to be high. A current multicenter study evaluates these findings longitudinally in a large cohort of FCs.

## Background

Family caregivers (FCs) are a pivotal source for quality of life, well-being and quality of care in terminally ill patients. However, they are also affected by caring for the patient and may thus be affected by significant physical and psychosocial burden [1, 2]. Therefore, the palliative care concept considers patients and their FCs as a “unit of care” [3]. The awareness of FCs’ concerns and needs, supply of supportive care and integration of FCs in the treatment and care planning represent important characteristics of palliative care [4].

Previous studies have demonstrated frequent physical health problems in FCs of home cared terminally ill patients, including exhaustion and sleep disturbances [5, 6]. At the social level, FCs are prone to financial problems, isolation, and occupation-related problems in the phase of caregiving [2, 7]. Psychological problems in FCs of patients undergoing palliative care are various with rates of anxiety and depression in up to 40% [2, 8], distress [8], signs of posttraumatic stress disorder [9] or emotional problems including helplessness, uncertainty, and hopelessness [10, 11]. Overall, FCs’ perceived burden and psychosocial concerns increase with the patients’ disease progression [10]. Various patient-related and socio-demographic risk factors for FCs burden as well as interactions between psychosocial problems are discussed [8, 12–15].

Psychosocial burden and anxiety are associated with the number of unmet needs in FCs of advanced cancer patients [16]. Frequently unmet needs of FCs relate to information on caregiving and care planning, support in managing fear as the patient’s physical or psychological status declines, and preparing for the patient’s death and their own bereavement [17–20]. Supportive needs can be summarized in four main categories: needs concerning the patient’s well-being, transfer of information, practical problems of caregiving, and emotional support [21]. Some studies suggest that FCs’ needs might be better addressed if specialized palliative care is included during home or hospital care [22]. In addition, FCs’ quality of life has been shown to be higher in patients receiving care in palliative care facilities rather than in acute care hospitals [23, 24]. Quality of life in FCs seems to be influenced by their satisfaction with quality of patient care, but also with their level of distress and other psychosocial factors [24–26]. The recognition and acknowledgement of FCs’ psychosocial burden by health care professionals, successful symptom management, supply of suitable information and adequate care planning are characteristics of higher quality of care [27, 28].

Although, research on FCs in palliative care has steadily increased over the last decade, a substantial body of literature focuses on home and ambulatory care, but does not specifically address the specialized inpatient palliative care (SIPC) setting. Studies on experiences of FCs of palliative patients in hospitals provide useful information about the range of potential psychosocial problems and needs. For example, in intensive care units, a substantial proportion of FCs showed symptoms of post-traumatic stress disorder (51%), anxiety (48%) or depression (33%) [29]. A study comparing FCs’ burden and quality of life in active treatment settings and hospice care reported the hospice group to have significantly lower values in the observed emotional and mental domains [30].

Assessing and responding to FCs’ psychosocial burden, specific problems and unmet needs are essential aims of SIPC. Results of studies from home-based palliative care have limited comparability and value for the specific characteristics of SIPC. FCs might benefit from medical around-the-clock services during SIPC, low-threshold psychosocial services available for both the patient and their FCs, and the possibility to opt for the degree of involvement in caring for the patient. On the other hand, hospital-based care might not be the patient’s and FCs’ preferred setting for end-of-life care. Therefore, sector-specific knowledge would help target services to those individuals who would benefit from early and preventive supportive care interventions during SIPC.

Primary aim of this pilot study was to address this gap in the literature for FCs of terminally ill cancer patients during SIPC. Therefore, we explored quality of life, psychological burden, unmet needs, and care satisfaction in FCs of advanced cancer patients in SIPC. Furthermore, we aimed to test feasibility and acceptance of the recruitment strategies and the applicability of a complex set of questionnaires in the vulnerable group of FCs during SIPC.

## Methods

### Study design and population

This single-center pilot study included a consecutive sample of FCs of advanced cancer patients admitted to the SIPC ward of a University Medical Center within a period of 12 weeks. Patients were admitted to the SIPC ward due to significant physical and / or psychosocial symptoms prohibiting further care at home or in non-specialized inpatient wards. We recruited FCs consecutively within 72 h after the patient’s admission to the SIPC ward. Inclusion criteria were being the primary FC as indicated by the patient – independent of biological or social relationship –, and being older than 18 years. Informed consent was obtained from all FCs included in the study. Exclusion criteria were a non-oncological disease of the patient, exclusively holding a legal guardianship for the patient, inadequate language skill to fill in the questionnaire, and insufficient cognitive function (physician’s assessment).

Data were collected by FC questionnaires at the beginning of the patient’s stay at the SIPC ward. The questionnaire and a prepaid return envelope were handed over by study staff. FCs could decide to either return the completed questionnaire to any member of the palliative care team or to return it by mail. In case of not having returned the questionnaire within 1 week, FCs received a one-time reminder through personal contact or by phone.

In order to prevent potential study-induced burden, FCs were asked to immediately inform the palliative care team in case of any problems and need for relief, and a trained psycho-oncologist was appointed as contact person. The local ethics committee of the General Medical Council of Hamburg approved the study protocol (PV5122, 08 December 2015).

### Measurements of outcomes

In order to assess FCs’ burden and needs, FCs completed a set of questionnaires consisting of the following validated scales (German versions):

Short Form-8 Health Survey (SF-8) [31, 32], Distress Thermometer of the National Comprehensive Cancer Network (DT) [33, 34] and adapted problem list, Generalized Anxiety Disorder 7-item scale (GAD-7) [35], Patient Health Questionnaire depression module 9-item scale (PHQ-9) [36], Family Inventory of Needs (FIN) [37, 38], Palliative Care Outcome Scale (POS) [39], Family Carer Satisfaction with Palliative Care scale (FAMCARE-2) [40, 41], and socio-demographic variables. Table 1 presents relevant information on the included scales. As for the abovementioned measurements, the applied questionnaire was of a complex nature, both in length (14 pages) and comprehensiveness (inclusion of multiple psychosocial and care-related scales).

**Table 1:** Measurements included in the study

Measure	Questionnaire	Description	Analysis and special aspects
Quality of life	Short Form-8 Health Survey (SF-8)	Validated short form of the Health survey Form-36 [31, 32]. Eight aspects of quality of life rated on six-point Likert scale ranging from 0 (excellent) to 5 (very bad). Higher values represent higher quality of life.	For the German version of the SF-8, age- and gender-specific data for the adult norm population are available [32].
Distress	Distress Thermometer (DT) of the National Comprehensive Cancer Network	Measures the subjective distress within the last week on an analogue scale ranging from 0 (no distress) to 10 (extreme distress) [33, 34]. An additional list of problems potentially causing this distress has to be answered with “yes” and “no”. The list was adapted and consisted of 23 problems.	For the German version, a cut-off value of ≥ 5 is recommended to detect clinically relevant distress with need of professional psychological support [34].
Anxiety	Generalized Anxiety Disorder 7-item scale (GAD-7)	Based on the DSM-IV diagnostic criteria for generalized anxiety disorder with excellent reliability and validity [35]. Frequency of core symptoms of generalized anxiety disorder within the past 2 weeks. Items are scored on a four-point Likert scale ranging from 0 (not at all) to 3 (nearly every day) with a total score ranging from 0 to 21.	A score up to 4 indicates the absence, scores of 5–9 mild, scores of 10–14 moderate and scores of ≥ 15 severe anxiety symptom levels [35]. Values for German standard population are available [43].
Depression	Patient Health Questionnaire depression module 9 (PHQ-9)	Based on the DSM-IV diagnostic criteria for depression with excellent reliability and validity [36]. The 9 items assess the frequency of depressive symptoms within the past 2 weeks. Items are scored on a four-point Likert scale ranging from 0 (not at all) to 3 (nearly every day) with a total score ranging from 0 to 27.	A score up to 4 indicates the absence, scores of 5–9 mild, scores of 10–14 moderate and scores of ≥ 15 severe depressive symptom levels. Values for German standard population are available [36].
Unmet needs	Family Inventory of Needs (FIN)	Measures the supportive needs of family caregivers and the extent to which these are met [37, 38]. 20 items are rated on two subscales: Subscales FIN–Importance and FIN–Fulfillment. Ratings between 1 (not important) and 5 (extremely important) for FIN–Importance and 0 (not met), 0.5 (partly met), and 1 (met) for the FIN–Fulfillment subscale.	For FIN-Importance, answer categories were dichotomized into “not/somewhat/moderate” vs. “very/extremely important”. For FIN-Fulfillment, indicated needs (at least “somewhat important”) were rated “unmet” if partly or not met. The German version was used with courtesy of Schur et al. [38].
Palliative care outcome	Palliative Care Outcome Scale (POS)	11 items for physical, practical, emotional, and psychosocial concerns of the patient and family caregiver; overall score ranges from 0 to 40 [39]. A lower sum score indicates better palliative care outcome.	Reference values from German validation in patients and professionals are available [39].
Satisfaction with palliative care	FAMCARE-2	Revised version of the FAMCARE tool used to measure family satisfaction with advanced cancer care. Consists of 17 items scored on a five-point Likert scale ranging from “very satisfied” to “very dissatisfied” [40, 41]. A total score ranging from 17 to 85 and four subscale scores can be calculated. Higher sum scores indicate higher satisfaction.	The German version was used with courtesy of Sewtz et al. [41].

To test for the feasibility and acceptance of the recruitments strategies and the applied questionnaire, we defined study participation, response rate and study-induced reference to a psycho-oncologist as relevant outcomes. Respective information was systematically collected throughout the study period by study staff. Based on the consecutive design, a documentation form was used to record occurrence and reasons of non-participation and drop-out. Furthermore, we followed up a checklist for the purpose to document all FCs who reported burden associated with study participation (and were therefore referred to the assigned psycho-oncologist).

### Statistical analysis

Descriptive analysis was used to examine sample characteristics, feasibility-related outcomes and FCs’ outcomes regarding quality of life (SF-8), psychological burden (DT, GAD-7, PHQ-9), supportive needs (FIN), and satisfaction with provision of palliative care (POS, FAMCARE-2). For analysis of multi-item questionnaire scales, we imputed missing data by using the mean, provided that less than two items were missing (individual level).

Furthermore, we conducted a series of statistical tests for group differences. We compared SF-8 scores to the scores of a German population-based sample [32] using one-sample t-test. For this analysis, we adjusted for age and gender by matching each FC with the value of a norm sample person from the same age and gender category.

Group comparisons of FCs’ outcomes based on age (< 60 vs. ≥ 60 years), gender and patient-caregiver relationship (spouse/partner vs. others) were performed by using chi-squared-test or Fisher’s exact test for categorical and two-sample t-test (two-tailed) for interval scaled variables. As the chi-square test assumes the expected value of each cell of the contingency table to be five or higher, Fisher’s exact test was used if this assumption was not met in our data.

We used Spearman’s rho and Pearson’s r to investigate bivariate associations of the level of satisfaction with care (FAMCARE-2) and the following variables: FCs’ age (< 60 vs. ≥ 60 years), gender, relationship to the patient (spouse/partner vs. others), level of education (elementary vs. junior high vs. high school), work status (currently working vs. not working), involvement in patient care prior to SIPC (yes vs. no), psychological burden (DT, GAD-7, PHQ-9), quality of life (SF-8) and FC’s needs (FIN). Furthermore, we calculated Pearson’s r to examine associations between the palliative care outcome (POS) and the ECOG performance status of the patient as assessed by the FC.

All significance tests were two-tailed using a significance level of α < 0.05. Analyses were performed using the statistical package SPSS Statistics software version 22.0 (IBM, USA).

## Results

### Feasibility of questionnaire survey

Within a study period of 12 weeks, 137 patients were admitted to the SIPC ward. Two of these patients were diagnosed with a non-oncological disease and eight did not or could not name a FC (13%). In 127 patients, a FC could be identified. Out of these 127 FCs, 22 could not be recruited due to the patient’s death within 72 h after admission (17%), 18 due to unavailability of the primary FC at the SIPC ward within 72 h (14%), and 13 due to language problems or insufficient cognitive function. Overall, 74 FCs were asked to take part in the study, of which 20 refused participation (27%). Finally, 54 FCs were willing to participate (73%), whereof 51 (94%) completed the administered questionnaire. Regarding the timeframe of response (calculated from the date of study inclusion and questionnaire completion), FCs answered the questionnaire on average within 3.5 days (SD 4.4). Of the three FCs who did not return the questionnaire (drop-out), the patient had died shortly after study inclusion.

Only one person was referred to the assigned psycho-oncologist because the FC expressed need for psychological support in the course of answering the questionnaire. Independent of the study, all FCs were cared for within the routine psychosocial support implemented within our SIPC.

### Family caregiver and patient characteristics

The mean age of the participating 51 FCs was 56 years (SD 15.5; range: 20–87), 55% of the sample were female (Table 2). The corresponding 51 advanced cancer patients had a mean age of 65 years (SD 13.6; range: 27–88). Patients were admitted from other hospital wards (36% of the cases), home care with nursing service (20%), home care with specialized palliative care service (36%) and nursing homes (8%). ECOG performance status was 4 in 62%, 3 in 26%, and ≤ 2 in 12% (mean 2.4, SD 0.86) of the patients. Out of 51 FCs, 22 reported to have cared for the patient before admission to the SIPC. The mean length of the caregiving period was 13.3 month (SD 14.4, range: 1–45) and the daily time spent on caregiving was 8.5 h (SD 8.6, range: 1–24 h).

**Table 2:** Family caregiver characteristics (*N* = 51)

		N FC	%
Age	< 60	32	63
	≥ 60	18	35
	Missing	1	2
Gender	Female	28	55
	Male	23	45
Relationship to the Patient. Patient is...	Spouse/partner	27	53
	Parent	23	26
	Child	3	6
	Sister/brother	6	12
	Friend	2	3
Marital status	Single	11	22
	Married	30	59
	Divorced/widowed	9	18
	Missing	1	2
Religious confession	Yes	32	63
	No	18	35
	Missing	1	2
Graduation	Elementary school (8–9 years)	15	29
	Junior high school (10 years)	14	28
	High school (12–13 years)	20	40
	Missing	2	3
Employment	Currently yes	24	47
	Currently no	25	49
	Missing	2	4
Involvement in patient care prior to SIPC	Yes	22	43
	No	29	57

*Abbreviations: N FC* number of family caregivers

### Quality of life and psychosocial burden

Except for the “bodily pain” scale, FCs reported significantly worse quality of life (SF-8) compared to a population-based reference sample on all subscales. The highest impairments were found for the “role emotional” and “mental health” scales with a difference of up to 10 points compared to the normalized mean of the German norm sample. FCs reported significantly impaired mental health sum scores (minus 10 points difference) compared to the norm sample, while the physical health sum scores did not differ from the reference sample (Table 3).

**Table 3:** Means of quality of life of family caregivers (*N* = 51) compared to German norm population (*N* = 2552)

Quality of life (SF-8)	Family caregiver pilot study population (*N* = 51)	Age- and sex-adjusted German population-based sample (*N* = 2552) [30]	Significance (one sample *t*-test)
Physical functioning	44.7 (SD 9.1, range 21.5–54.1)	47.7 (SD 2.5)	***p*** **= 0.022** ^*)^
Physical role	44.3 (SD 9.1, range 23.0–54.0)	48.3 (SD 2.2)	***p*** **= 0.003** ^*)^
Bodily pain	52.0 (SD 10.1, range 25.5–61.0)	52.6 (SD 2.3)	*p* = 0.654
General health	42.2 (SD 7.1, range 22.8–59.5)	47.1 (SD 2.2)	***p*** **< 0.001** ^*)^
Vitality	47.0 (SD (8.0, range 35.8–61.8)	51.0 (SD 2.4)	***p*** **= 0.001** ^*)^
Social functioning	45.1 (SD 9.8, range 23.4–55.3)	50.6 (SD 1.2)	***p*** **< 0.001** ^*)^
Emotional role	38.3 (SD 9.8, range 21.7–52.4)	48.6 (SD 1.1)	***p*** **< 0.001** ^*)^
Mental health	42.4 (SD 10.2, range 21.4–56.8)	52.3 (SD 0.9)	***p*** **< 0.001** ^*)^
Physical sum score	46.8 (SD10.5, range 18.0–61.1)	48.7 (SD 3.2)	*p* = 0.206
Mental sum score	40.9 (SD 9.6, range 16.3–56.1)	52.8 (SD 1.1)	***p*** **< 0.001** ^*)^

*Abbreviations: SD* standard deviation

^*)^ = statistically significant *p* < 0.05

FCs of higher age (≥ 60 years) were found to have significantly lower physical functioning (*p* = 0.004) and lower values of the physical sum score (*p* = 0.026), while gender or patient-caregiver relationship had no impact on quality of life outcomes.

Psychosocial distress (DT, scale 0–10) was clinically relevant (values ≥ 5) in almost all FCs (49 of 51 / 96%), while severe distress (values ≥ 8) was reported by 26 FCs (51%). Out of 23 given problems of the adapted problem list, sadness (83%), sorrows (80%), exhaustion (80%), and anxiety (70%) were the four most prevalent issues indicated by FCs (Table 4).

**Table 4:** Results of the adapted problem list of the Distress Thermometer used in family caregivers (*N* = 51)

Problem present:	N FC	%
Practical problems:		
Living situation:	6/45	13
Insurance:	8/44	18
Transportation/mobility:	5/43	12
Employment/school:	9/40	23
Child care:	3/39	8
Financial problems:	5/43	12
Family and social problems:		
Relationship with partner	5/41	12
Relationship with children	6/40	15
Relationship with friends	7/43	17
Emotional problems:		
Sorrows	37/46	80
Anxiety	30/43	70
Sadness	39/47	83
Depression	8/39	21
Nervousness	20/43	47
Isolation	12/40	30
Spiritual problems:		
Loss of trust	2/36	6
Spiritual aspects concerning god	4/38	11
Physical problems:		
Pain	10/44	23
Exhaustion	39/49	80
Sleeping problems	31/45	69
Digestion problems	8/43	19
Problems with eating/diet	14/44	32
Cognitive problems/concentration	25/47	53

*Abbreviations: N FC* number of family caregivers

Anxiety (GAD-7) was absent in 11 FCs (23%), mild in 17 (33%), moderate in 16 (31%), and severe in six (12%; missing *n* = 1). The mean GAD-7 score in FCs was 9.0 (SD 5.0, range: 0–21). Depression (PHQ-9) was absent in 13 FCs (26%), mild in 17 (33%), moderate in 15 (29%) and severe in six (12%). The mean PHQ-9 score was 8.6 (SD 4.9, range: 0–21).

Based on gender, age and patient-caregiver-relationship we found no group differences regarding distress, anxiety or depression (all *p* > 0.1).

### Unmet needs, perceived palliative care outcome, and satisfaction with care

FCs scored a mean number of 16.3 of 20 caregiver-specific needs to be very or extremely important (FIN-Importance scale; SD 3.3, range 2–20). Nine needs (45%) were rated as very important by more than 90% of the FCs, and 14 needs (70%) were reported to be extremely important in more than 80%. As for the fulfillment of needs, a mean number of 13.7 needs that were rated to be at least somewhat important were satisfied (FIN-Fulfillment scale; SD 5.2, range 2–20) (Table 5). Four needs (20%) were unmet in more than 50% of FCs who reported this need at least to be somewhat important (54–68%). These were “feel there is hope” (68%), “know when to expect symptoms to occur” (57%), “have somebody be concerned with my health” (54%), and “be told about people who could help with problems” (56%). The mean number of very or extremely important needs was significantly higher in older patients (≥ 60 years; *p* = 0.025), but independent from gender and patient-caregiver relationship. Regarding the mean number of fulfilled needs, no group differences occurred (all *p* > 0.1).

**Table 5:** Results of the needs assessment, palliative care outcome, and satisfaction with care in family caregivers (*N* = 51)

	Mean	SD
Supportive Needs (FIN)		
Number of needs with high importance	16.3	3.3
Number of needs met^*)^	13.7	5.2
Palliative care outcome – sum score (POS)	16.6	5.0
Satisfaction with care (FAMCARE-2)		
FAMCARE-2 total score	73.4	8.4
Physical symptoms and comfort	22.4	2.6
Information	16.5	2.2
Family support	17.0	2.4
Patient psychological care	17.5	2.6

*Abbreviations: SD* standard deviation

^*)^based on all needs rated to be at least “somewhat important”

Mean POS sum score was 16.6, indicating a good quality of patient care from the FCs’ perspective (SD 5.0, range 5–28; 0–40 points possible) (Table 5). Subjective perception of the palliative care outcome significantly correlated with the patient’s ECOG performance status (assessed by the FC, *p* = 0.020), but no group differences regarding age, gender or patient-caregiver relationship were observed.

Overall satisfaction with palliative care (FAMCARE-2 total score) was high with 73.4 points in mean (SD 8.4, range: 51–85; 17–85 points possible). FCs reported high satisfaction means across all subscales: 22.4 points for physical symptoms and comfort (SD 2.6, range 14–25; scale: 5–25), 16.5 for information (SD 2.2, range 10–20; scale: 5–20), 17.0 for family support (SD 2.4, range: 12–20; scale: 5–20), and 17.5 for patient psychological care (SD 2.6, range: 8–20; scale 5–20) (Table 5). Overall satisfaction with palliative care was significantly higher in female FCs (*p* = 0.037), but independent of other socio-demographic characteristics, psychological burden (DT, GAD-7, PHQ-9), quality of life (SF-8) or the importance and fulfillment of needs (FIN, all *p* > 0.05).

## Discussion

This single-center pilot study demonstrates the feasibility of conducting a questionnaire survey in FCs of advanced cancer patients in the specific setting of SIPC using a complex set of measurements.

Overall, 73% of the eligible FCs were willing to participate in the study whereof 94% completed and returned the questionnaire. The high study commitment of FCs is in line with results of Stiel et al. who demonstrated that bereaved FCs after palliative care are willing to support end-of-life care research and partially even report benefits from study participation [42].

Previous studies demonstrated anxiety and depression rates of up to 40% in FCs of palliative care patients [2]. Similarly, in our cohort, 43% and 41% of FCs reported moderate or severe anxiety and depression, respectively. With respect to the limited sample size and no age- and gender-specific matching of data, any comparisons have to be taken with caution, but anxiety seemed to be more intensive in FCs than in healthy German people (mean scores of 9.0 vs. 3.0 in GAD-7) [43]. Furthermore, our findings might suggest depression to be more frequent in FCs than in a German norm sample, with rates of 25% vs. 76% for absence of depressive symptoms [36].

We used the Distress Thermometer [33, 34], which was primarily developed and validated in cancer patients, but also has been found to be a suitable tool for measuring distress in FCs of cancer patients [44, 45]. Almost all FCs (96%) reported clinically significant distress, scoring higher than the recommended cut-off (≥ 5). Psychosocial burden among FCs was substantial, since about half of the FCs reported distress scores of eight or higher. More than two thirds of FCs indicated sadness, sorrows, exhaustion, anxiety, and sleeping problems as relevant causes for their distress.

Based on the PHQ-9, approximately 74% of FC showed depressive symptoms of varying severity. In the adapted problem list (DT), 21% of FC indicated depression to cause distress. These results emphasize the relevance of distinguishing between the constructs of (i) depression as a potential reason for feeling distressed (observed by the FC) and (ii) the presence and severity of depressive symptoms assessed by a measure with diagnostic validity.

In our study, quality of life was worse in FCs compared to a sex- and age-adjusted German norm population [31], in particular in the mental health domain of quality of life. A recent study investigating quality of life in FCs of newly-diagnosed patients with incurable cancer found caregiver’s mental health to be lower and physical health to be better than the U.S. population normalized mean [15]. While findings of previous studies indicated caregiver’s quality of life to be associated with their relationship to the patient [15, 46], we found no such correlation in our study. Furthermore, studies suggested that FCs’ quality of life is influenced by their satisfaction with quality of patient care [24–26]. Interestingly, despite of the impaired quality of life of FCs in our sample, satisfaction with palliative care of the patients was high across all subscales and we found no associations between FCs’ satisfaction with patient care and their own quality of life. This lacking statistical effect might be caused by the limited sample size, but could also be associated with the generally high satisfaction during SIPC in our study. In previous studies, higher satisfaction with care was described in FCs of patients receiving specialized compared to general palliative care [22]. It should be noticed that this generally high satisfaction might be biased by social desirability during the patients’ stay on the SIPC ward and the dependency on inpatient palliative care. In our pilot study, satisfaction with palliative care provision was higher in female FCs, but unaffected by other socio-demographic variables, psychosocial factors as well as the importance and fulfillment of needs.

Overall, our data emphasize that many FCs suffer from relevant impaired quality of life and decreased psychosocial well-being during SIPC. FCs are affected by the imminent loss of a loved one, and our results might reflect feelings that represent an adequate response to the situation. On the other hand, across most outcomes, the degree of burden (in terms of scale range) differentiated among our cohort indicating FCs’ diversity of perceptions and suffering. This implies a careful examination of how to respond to FCs burden in terms of acknowledging the appropriateness and normality of feeling burdened, but also to assume responsibility for identifying FCs’ in need for professional support and for providing FC-directed interventions.

The Palliative Care Outcome Scale is frequently used as a routine assessment tool in specialized palliative care in Germany to measure the patient’s and palliative care staff’s perception of care outcome [39]. FCs rated the palliative care outcome with a mean sum score of 16.6, which indicates that the FCs’ perception of and satisfaction with care during SIPC is positive despite of their psychosocial burden. In previous studies in Germany, patients and palliative care staff have rated outcome with lower mean sum scores ranging between 11.9 and 15.9 [39].

Previous studies have demonstrated that psychosocial burden in FC of advanced cancer patients is associated with the number of unmet needs [11]. In our sample, 20% of needs were unmet in a majority of FCs. The most frequently unmet needs relate to hope, knowledge about when symptoms will occur, to have someone caring for the FC’s own health, and information about support. These results correspond with findings of a systematic review indicating that frequently unmet needs in FCs during palliative care are often aligned with lack of information [2].

Our findings raise the question if FCs might refrain from asking for meeting specific needs due to lacking knowledge or missing understanding of the palliative care principle of the “unit of care”. Moreover, FCs in palliative care might withhold needs from health care professionals. For example, a review on challenges for health care professionals to meet supportive needs of FCs in palliative care suggests that FCs may prioritize the patient’s needs in relation to their own, or may not want to bother health care professionals [47].

Interestingly, we did not find any associations between FCs’ unmet needs and their satisfaction with patient care. This finding might suggest that in the specific period of SIPC, FCs are able to distinguish their own needs both from the overall care situation and the patient’s needs. There is growing evidence on the concept of relational autonomy of patients in palliative care and their FCs, meaning that they suffer in relation to each other [48]. Therefore, FCs should be encouraged by health professionals to carefully observe their own needs and to seek for support within SIPC, both in view of the principle of “unit of care” as well as the interdependence of patient’s and FC’s burden.

In addition to the fulfillment of needs, the subjective importance of needs was assessed. In previous studies, the FIN scores “not/somewhat important” were compared with “moderate/very/extremely important” [37, 38]. Due to very low combined rates of “not and little important” needs (< 3% across the 20 given needs) in our sample, we dichotomized needs into “very and extremely important” and “not/little/moderate important” needs. Using this modified strategy, an impressive percentage of 80% of needs was rated as very or extremely important, and almost half of the given needs were rated as very or extremely important in more than 90% of FCs. Our results suggest show that FCs have many needs in a period in which the patient is cared for in the SIPC, with older FC reporting more needs.

To estimate the extent to which age, gender and patient-caregiver-relationship might affect FCs’ burden and needs, we systematically compared respective subgroups. In our study, we did not find any of these factors to be a clear and consistent gradient in FCs’ psychosocial burden, needs, and satisfaction with palliative care during SIPC. Due to the small sample size, we dichotomized the respective factors. Regarding the patient-caregiver-relationship, it should be noticed that a relatively large proportion of FCs were spouses/partners (53%) or children (26%) of the patients. It might be assumed that in comparison to other relatives, these groups of FCs are most affected by the imminent loss of the patient. Nevertheless, only half of our cohort was involved in caring for the patient before the admission to the SIPC. Irrespective of this rather moderate number of caregiving FCs and takeover of professional caring at the time of assessment, the burden of our FCs was high.

Overall, it has to be strengthened that these preliminary results have to be interpreted carefully due to the limited number of 51 included FC. They need to be interpreted as hypothesis generating and will be prospectively evaluated in a larger cohort.

## Conclusions

In conclusion, FC of terminally ill cancer patients seem to suffer from relevant psychosocial burden, including distress, anxiety and depression during SIPC. FCs’ quality of life seems lower than in the German norm population, particularly in the mental health domain. In addition, they report a significant number of unmet needs. These findings indicate the importance of regular assessment of psychosocial burden, quality of life and supportive needs in FC in routine specialized inpatient palliative care. However, FC’s satisfaction with SIPC is high and palliative care outcome seems to be regarded positive. This pilot study has demonstrated the feasibility of this complex questionnaire analysis and we generated first results on FC’s situation during SIPC. Nevertheless, FC’s psychosocial burden, needs, and coping strategies might change over time and thus should be measured repeatedly from the onset of the palliative disease through to bereavement. Our findings underline the importance of identifying FC’s at risk for high psychosocial burden and unmet needs in order to reduce the risk of strain and dysfunction.

## Abbreviations

DT: Distress Thermometer of the National Comprehensive Cancer Network
ECOG: Eastern Co-operative Oncology Group Performance Status
FAMCARE-2: Family Carer Satisfaction with Palliative Care scale
FC: Family caregiver
FIN: Family Inventory of Needs
GAD-7: Generalized Anxiety Disorder 7-item scale
PHQ-9: Patient Health Questionnaire depression module 9-item scale
POS: Palliative Care Outcome Scale
SF-8: Short Form-8 Health Survey
SIPC: Specialized inpatient palliative care
